# Cell Type-Dependent Activation Sequence During Rhythmic Bursting in the PreBötzinger Complex in Respiratory Rhythmic Slices From Mice

**DOI:** 10.3389/fphys.2018.01219

**Published:** 2018-09-03

**Authors:** Yoshihiko Oke, Fumikazu Miwakeichi, Yoshitaka Oku, Johannes Hirrlinger, Swen Hülsmann

**Affiliations:** ^1^Division of Physiome, Department of Physiology, Hyogo College of Medicine, Nishinomiya, Japan; ^2^Department of Statistical Modeling, The Institute of Statistical Mathematics, Tachikawa, Japan; ^3^Department of Statistical Science, School of Multidisciplinary Sciences, The Graduate University for Advanced Studies (SOKENDAI), Tachikawa, Japan; ^4^Carl-Ludwig-Institute for Physiology, Faculty of Medicine, University of Leipzig, Leipzig, Germany; ^5^Department of Neurogenetics, Max Planck Institute of Experimental Medicine, Göttingen, Germany; ^6^Clinic for Anesthesiology, University Medical Center Göttingen, Göttingen, Germany; ^7^Research Center for Nanoscale Microscopy and Molecular Physiology of the Brain, University Medical Center Göttingen, Göttingen, Germany

**Keywords:** preBötzinger complex, inspiratory neuron, inhibitory neuron, rhythmic burst, activation sequence, activation timing, calcium imaging

## Abstract

Spontaneous respiratory rhythmic burst activity can be preserved in the preBötzinger Complex (preBötC) of rodent medullary transverse slices. It is known, that the activation sequence of inspiratory neurons in the preBötC stochastically varies from cycle to cycle. To test whether the activation timing of an inspiratory neuron depends on its neurotransmitter, we performed calcium imaging of preBötC neurons using double-transgenic mice expressing EGFP in GlyT2^+^ neurons and tdTomato in GAD65^+^ neurons. Five types of inspiratory neurons were identified using the fluorescence protein expression and the maximum cross-correlation coefficient between neuronal calcium fluctuation and field potential. Regarding the activation sequence, irregular type putative excitatory (GlyT2^-^/GAD65^-^) neurons and irregular type glycinergic (GlyT2^+^/GAD65^-^) neurons tended to be activated early, while regular type putative excitatory neurons, regular type glycinergic neurons tended to be activated later. In conclusion, the different cell types define a general framework for the stochastically changing activation sequence of inspiratory neurons in the preBötC.

## Introduction

The preBötzinger Complex (preBötC) is the crucial kernel to generate the spontaneous respiratory rhythm and its activity can be preserved and recorded as inspiratory rhythmic bursts in a medullary transverse slice preparation (Smith et al., 1991; Feldman and Del Negro, 2006). Detailed knowledge of the activation sequence of different types of respiratory neurons is critical for the understanding of respiratory rhythmogenesis in the preBötC. In the rhythmic slice preparation, the activation sequence of inspiratory neurons changes stochastically with every respiratory cycle (Carroll and Ramirez, 2013; Oke et al., 2015). Furthermore, inspiratory neurons are rhythmically activated during rhythmic bursts with loose regularity, where a subset of inspiratory neurons appears to cover the initial part of the activation sequence (Oke et al., 2015). At present, regularities that control the stochastically changing activation sequence of inspiratory neurons are unrevealed. The sequential process of rhythmic burst generation can be decomposed into two processes (Kam et al., 2013; Del Negro et al., 2018): At first, preinspiratory activity of a subset of neurons produces a low amplitude signal [recorded as burstlet in the local field potential (LFP)], which determines initiation of the rhythm (rhythm generation). This activity produces the high-amplitude inspiratory LFP bursts, which determine the pattern of rhythmic network output, e.g., the activity of motoneurons. In recent *in vivo* experiments, optogenetics together with pharmacological perturbation revealed that various molecularly defined types of respiratory neurons, e.g., Dbx1^+^, SST^+^, VGAT^+^, and GlyT2^+^, play different roles in the sequential process of rhythm generation (Sherman et al., 2015; Cui et al., 2016; Baertsch et al., 2018). However, the role played by the diverse neuron types in generating the stochastic activation sequence of inspiratory rhythmic burst in the preBötC remained uninvestigated. In this study, we hypothesized that the neuron type defines a framework for the stochastically changing activation sequence of inspiratory neurons during inspiratory rhythmic bursts in the preBötC. To test this hypothesis, we investigated the activation sequence among glycinergic, GABAergic and putative excitatory neurons using double-transgenic mice (double-TG-mice) expressing EGFP in glycinergic neurons under the control of the GlyT2-promoter (GlyT2^+^ neurons) neurons (Zeilhofer et al., 2005) and tdTomato in GABAergic neurons under the GAD65-promoter (GAD65^+^ neurons) (Besser et al., 2015). Additionally, these cell types were functionally classified into regular type (R-) or irregular type (Irr-) based on the maximum normalized cross-correlation coefficient (maxCC) between the bursting pattern of integrated LFP and the fluctuation of intracellular calcium levels in inspiratory neurons. Based on our results, we propose a model for synaptic interactions among the different types of inspiratory neurons, which describes the generation of spontaneous inspiratory rhythmic bursts in the preBötC.

## Materials and Methods

### Animal Care and Breeding of Mice

This study was carried out in accordance with the guidelines for the welfare of experimental animals issued by the European Communities Council Directive 2010/63/EU and with the German Protection of Animals Act (TierSchG). Protocols (§4 Abs. 3 TierSchG) were approved and registered (T12/11) by the animal welfare office and commission of the University Medical Center Göttingen. Mice were bred in the animal facility of the University Medical Center Göttingen. We crossbred Tg(Gad2-tdTomato)DJhi-mice expressing red fluorescent protein tdTomato in GABAergic neurons (Besser et al., 2015) to Tg(Scl6a5-EGFP)1Uze-mice expressing green fluorescent protein EGFP in glycinergic neurons (Zeilhofer et al., 2005).

### Preparation for Rhythmic Slices

Rhythmic slices were prepared from mice between postnatal day 3–8 as described previously (Hulsmann et al., 2000; Winter et al., 2009). We decapitated the mice under isoflurane anesthesia and isolated the brainstem in ice-cold, oxygenated (95% O_2_, 5% CO_2_) artificial cerebrospinal fluid (aCSF) composed of (in mM): 118 NaCl, 3 KCl, 1.5 CaCl_2_, 1 MgCl_2_, 1 NaH_2_PO_4_, 25 NaHCO_3_, 30 D-glucose (pH 7.4). The brainstem was fixed to an agar block with needles (φ: 0.1 mm, Fine Science Tools Inc., North Vancouver, BC, Canada) and cyanoacrylate glue (Loctite Deutschland GmbH, Munich, Germany). Then, the agar was mounted on the plate of vibroslicer (Leica VT 1200S, Leica Biosystems, Nussloch, Germany) with the caudal end up and the ventral face toward the blade. Then, transverse slices (550–600 μm thickness), with exposing the preBötC on cutting plane of the rostral side, were prepared from the brainstem in ice-cold, oxygenated high osmolality ringer solution without calcium containing (in mM): 124 NaCl, 3 KCl, 2 MgCl_2_, 1.3 NaH_2_PO_4_, 26 NaHCO_3_, 10 D-glucose, 200 sucrose, 1 kynurenic acid (Richerson and Messer, 1995). For calcium imaging, the slices were transferred into the recording chamber that was mounted on an upright microscope (Axioscope FS, Zeiss, Germany) and were superfused in aCSF with 8 mM KCl at a flow rate of 4 ml/min at 28°C in order to induce rhythmic activity (Smith et al., 1991).

### Staining for Calcium Imaging

For calcium imaging, the preBötC was stained by Oregon Green 488 BAPTA-1 AM (OGB-1, Thermo Fisher Scientific Inc., Waltham, MA, United States) as described in detail previously (Winter et al., 2009). Briefly, 50 μg of OGB-1 was dissolved in 40 μl of DMSO containing 20% Pluronic F-127 (Thermo Fisher Scientific Inc., Waltham, MA, United States), and stored at -20°C in 4 μl aliquots before use. One aliquot of this stock solution was dissolved in 16 μl of an extracellular solution containing the following (in mM): 150 NaCl, 2.5 KCl, 10 HEPES, pH 7.4) to prepare 200 μM of OGB-1 at a final concentration. The OGB-1 solution was injected into the preBötC at the depth of 50–100 μm from the rostral surface of the slice for 10 min under 0.7 bar, followed by a perfusion for >40 min at 28°C to washout excess OGB-1.

### Calcium Imaging Using 2-Photon Microscopy

Imaging was performed with 2-photon laser-scanning microscope (TriMScope, LaVision, BioTec, Bielefeld, Germany) using 20× (1.0 NA) water immersion objective lens (Zeiss, Oberkochen, Germany) and GaAsP photomultipliers for non-descanned detector (Hamamatsu Photonics K.K., Hamamatsu, Japan). Two photon excitation was achieved with a Ti:Sapphire Laser (MaiTai BB, SpectraPhysics, Santa Clara, CA, United States). Before calcium imaging, reference images for EGFP (in glycinergic neurons), tdTomato (in GABAergic neurons) and OGB-1 (in all cells) were obtained as a size of 500 × 500 pixels (= 250 μm × 250 μm) by averaging 5× or 10× scanning of each line at 1,400 Hz per line. For excitation three wavelengths (720, 800, or 900 nm) were used. Emitted fluorescence was simultaneously detected through three kinds of band-pass emission filters (641/75, 531/40, or 475/50 nm). Spectra overlapping of fluorescence was decomposed off-line (see the section “Image Processing”). For calcium imaging, OGB-1 fluorescence was detected through a 531/40 nm band-pass emission filter with excitation at 800 nm wavelength. Each frame for a series of calcium imaging was captured as a size of 256 × 256 pixels (= 250 μm × 250 μm) at about 10 Hz by single line scanning at 3,000 Hz per line. The sampling frequency slightly differed for each imaging data. Optical filters were obtained from AHF Analysentechnik AG (Tübingen, Germany). All settings were controlled by “Imspector” software (LaVision, BioTec, Bielefeld, Germany).

### Electrophysiological Recording

Glass electrodes were filled with aCSF for recording. LFP was amplified 5,000–200,000 times, band-pass filtered (0.25–3.5 kHz) and digitized at 10 kHz on Digidata interface using pClamp10 software (Molecular Devices Inc., San Jose, CA, United States). The electrical signals were rectified and integrated (time constant = 100–200 ms) online using a custom-made amplifier (Electronic workshop, Physiology, Göttingen). The trigger pulse for each image frame was recorded simultaneously with the LFP signal for later off-line analysis. LFP data were resampled at the same sampling rate of calcium imaging data using an anti-aliasing method with a low-pass Chebyshev Type I infinite impulse response (IIR) filter of order 8, which is implemented in MATLAB.

### Image Processing

**FIGURE 1:**
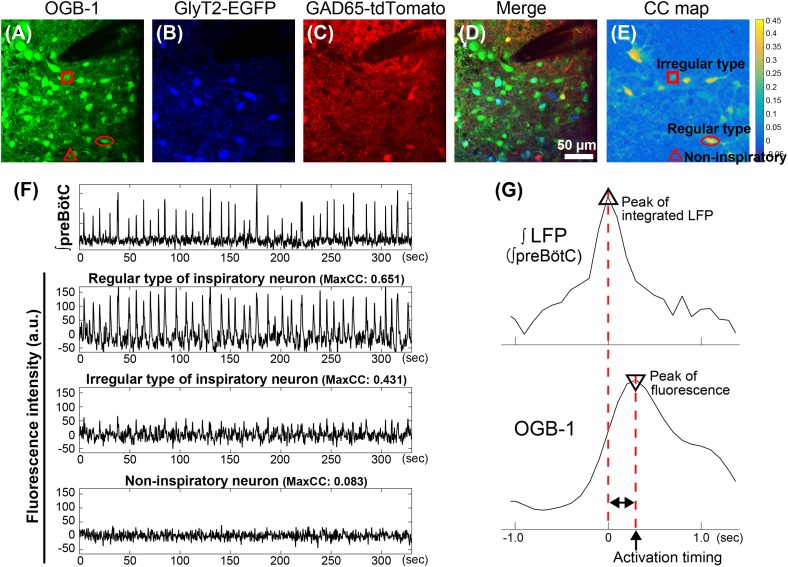
Classification and analysis of inspiratory neurons in the preBötC in GlyT2-EGFP and GAD65-tdTomato double-transgenic-mice. **(A–D)** Fluorescence images in the preBötC in the transverse slice from GlyT2-EGFP and GAD65-tdTomato double-TG-mice. Images were calculated to separate the spectrally overlapping fluorescence from EGFP, tdTomato and OGB-1 using non-negative tensor factorization. **(A)** Injection of OGB-1, calcium indicator, visualized cells in the preBötC. **(B,C)** EGFP (blue) and tdTomato (red) were observed in GlyT2^+^ glycinergic neurons and GAD65^+^ GABAergic neurons, respectively. **(D)** Overlay image of **(A–C)**. Glycinergic neurons (GlyT2^+^/GAD65^-^ neuron), GABAergic neurons (GlyT2^-^/GAD65^+^ neuron), or GlyT2^+^ and GAD65^+^ double positive, potential dual transmitting neurons (Cotrans neurons) were shown as blue, red, and yellow cells, respectively. Inspiratory neurons without expression of EGFP and tdTomato are referred to as excitatory neurons (green). Dark area at upper right reflects the position of the glass pipette for recording LFP. **(E)** Cross-correlation image was calculated between fluctuations of OGB-1 fluorescence at individual pixels and the waveform of integrated LFP. **(F)** Traces show a typical integrated LFP and simultaneous fluctuations of OGB-1 fluorescence on neurons (arbitrary units). Fluorescence intensities on regular and irregular types of inspiratory neurons and non-inspiratory neuron were measured in ROIs of 7 × 7 pixels (6.8 μm × 6.8 μm) on neurons indicated by circle, square and triangle in **(A,E)**, respectively. Regular or irregular type was classified based on maxCC between fluctuation of OGB-1 fluorescence and bursting pattern in integrated LFP. **(G)** Scheme illustrates the procedure to determine an activation timing of an inspiratory neuron during a rhythmic burst. Upper and lower traces show enlarged integral LFP and simultaneous OGB-1 fluorescence fluctuation from a regular type of excitatory neuron, respectively. Activation timing is defined as a time difference calculated by a peak of OGB-1 fluorescence intensity from a peak of an integrated LFP.

All “Imspector”-images were exported to TIFF format and preprocessed by Image-J^1^. Considering spectral overlapping of fluorescence from OGB-1, EGFP, and tdTomato, we used a spectral unmixing plug-in for Image-J to separate the signal from the three fluorophores using non-negative tensor factorization (Neher et al., 2009). For solid decomposition, we recorded stacks of fluorescence images using the three band-pass filter sets for three different excitation wavelength, allowing classification of glycinergic neurons expressing EGFP, GABAergic neurons expressing tdTomato and dual-transmitting neurons expressing both fluorophores. All further signal processing and detection of activation timings were done using MATLAB (The Math Works, Natick, MA, United States) as slightly modified the previous method (Boiroux et al., 2014). In order to improve signal to noise ratio, we applied spatio-temporal filtering to series of calcium imaging as follows. First, the time series corresponding to respective image pixels were band-pass filtered (0.025–1.5 Hz, third order zero-phase Butterworth filter). Then, the data were filtered spatially by taking the unweighted average of a 3 × 3 region around each pixel. Cross-correlation image was made by calculation of the maximum normalized cross-correlation coefficient (maxCC) between bursting pattern of integrated LFP and fluctuation of OGB-1 fluorescence at each pixel (**Figure 1E**). Maxima of the cross-correlation coefficient were searched within a time lag between 0 and 40 frames. A region of interest (ROI) of 7 × 7 pixels was set on a position of cell indicating a maxCC greater than a preset threshold (maxCC = 0.2). We visually double-checked whether peaks of fluorescence fluctuations in ROIs had been coincident with rhythmic burst signals in the integrated LFP.

### Discrimination of Regular Type or Irregular Type of Inspiratory Neurons

We classified inspiratory neurons into “Regular type” and “Irregular type” neurons using the maxCC as a reference. A cut-off maxCC was adjusted empirically for each experiment (range from 0.47 to 0.64) in the individual experiment because maxCC depended on many factors, e.g., the number of bursts, noise levels, lengths of recordings which were different among individual slices. Cells with maxCC above the cut-off were called “Regular type.”

### Determination of Activation Timing

We set the peak of integrated LFP during individual rhythmic bursts as (*t* = 0). In order to exclude small fluctuations (noise and burstlet), we detected a peak of rhythmic burst only when a peak value of the integral LFP was larger than 0.8× standard deviation of the detrended LFP signal, which was decimated at the same sampling rate as the correspondent calcium imaging. Every increase of OGB-1 fluorescence above noise [in a 7 × 7 pixel region of interest (ROI)], was defined as an “activation” occurrence in a inspiratory neuron, since the size of OGB-1 signal depends on the calcium ion concentration, which in turn depends on the number of action potentials (Winter et al., 2009). Activation timing (in relation to the peak of the LFP) was defined as the time of the OGB-1 fluorescence peak in a window of 24 frames before and after (*t* = 0). (**Figure 1G**). Timing was automatically detected by the MATLAB^®^ macro but always visually confirmed afterward. Moreover, activation timings of individual inspiratory neurons were only defined for those cycles in which at least 50% of all inspiratory neurons were activated, to (1) exclude burstlets from our analysis and (2) to minimize errors about the given position of cells in the activation sequence that result from the different number of the activated cells per cycle. We also removed sigh-like big rhythmic bursts from the analyses. During a sigh-like big rhythmic burst, repeated calcium fluorescent peaks frequently appeared, which made it difficult to define the activation timing during a sigh-like burst (data not shown).

### Analysis of Activation Order in the Sequence

For every respiratory cycle, neurons were sorted in the order of the activation, to define their position in the activation sequence. The “number of activations” at a given position in the sequence was calculated for each cell type. Since this number of position depends on the number of inspiratory neurons in a slice (Slice1, 2, 3, 4, 5; *n* = 16, 17, 14, 22, 15 neurons), we converted the given position into a percent rank (bin size 10%) to allow statistical analysis.

### Statistical Analysis

One-way ANOVA were conducted for all statistical comparison among five cell types except the statistical comparison of the cumulative distributions. For one-way ANOVA, *post hoc* significance for multiple comparisons was analyzed using Tukey’s test. The statistical comparison of the cumulative distributions with pairwise Kolmogorov–Smirnov test were performed to investigate the difference of distributions. Data are shown as mean ± SE in graphs.

## Results

### Classification of GlyT2^+^, GAD65^+^ or Excitatory Neurons in the PreBötC Using Double-TG-Mice

To classify inspiratory neurons in the preBötC, we prepared rhythmic slices from GlyT2-EGFP and GAD65-tdTomato double-TG-mice (**Figure 1A**). As previously reported (Mesuret et al., 2018), GlyT2^+^ glycinergic neurons or GAD65^+^ GABAergic neurons could be visually discriminated by expression of EGFP (**Figure 1B**; blue) or tdTomato (**Figure 1C**; red), respectively. GlyT2^+^/GAD65^+^ neurons expressing both two fluorescent proteins are expected to be dual transmitting and appear as yellow cells in **Figure 1D**. Further, we considered respiratory rhythmic cells showing only OGB-1 fluorescence (without expression of any fluorescent proteins) as putative excitatory neurons (**Figure 1D**; green).

### Pattern-Dependent Classification of Inspiratory Neurons in the PreBötC

Fluctuation of somatic intracellular calcium concentration indicates a consequence of action potential firing during rhythmic bursts. Based on the fluctuation patterns of OGB-1 fluorescence, inspiratory neurons in the preBötC were further classified into two groups, “Regular type” and “Irregular type.” For this classification, we used time-lagged maxCC as a parameter. The maxCC is an index value how the full shape of OGB1 fluorescence waveform resembles the LFP waveform. The magnitude of the maxCC was mainly affected by the pattern of OGB-1 fluorescent fluctuation, e.g., whether the activation of the neuron occurred every cycle accurately, whether there were no activation occurrences between rhythmic bursts, and whether time-differences between the peak of LPF and the peak of OGB-1 fluorescence were uniform cycle-by-cycle. “Regular type” (R-) inspiratory neuron showed a large maxCC and exhibited clear waveforms of intracellular calcium rises during rhythmic bursts (**Figure 1F**, second trace from top). “Irregular type” (Irr-) of inspiratory neuron, had a small maxCC (cut-off maxCCs: range from 0.47 to 0.64: see the section “Discrimination of Regular Type or Irregular Type of Inspiratory Neurons”) and its fluctuation of intracellular calcium level were rather small and short (**Figure 1F**, third trace from top). Together with the expression of fluorescent proteins, this allowed us to discern five different types of inspiratory neurons: Two groups of regular type neurons; (a) GlyT2^-^/GAD65^-^, putative excitatory (R-Ex) and (b) GlyT2^+^/GAD65^-^, glycinergic (R-Gly) neurons and three groups of irregular type neurons; (i) GlyT2^-^/GAD65^-^, putative excitatory (Irr-Ex) (ii) GlyT2^+^/GAD65^-^, glycinergic (Irr-Gly), and (iii) GlyT2^+^/GAD65^+^, dual transmitting (Irr-Cotrans) neurons in the preBötC using four rhythmic slices. Other types of inspiratory neurons (R-GlyT2^-^/GAD65^+^, Irr-GlyT2^-^/GAD65^+^, R-GlyT2^+^/GAD65^+^) were not detected.

### Activation Timing of Individual Cell Types

**FIGURE 2:**
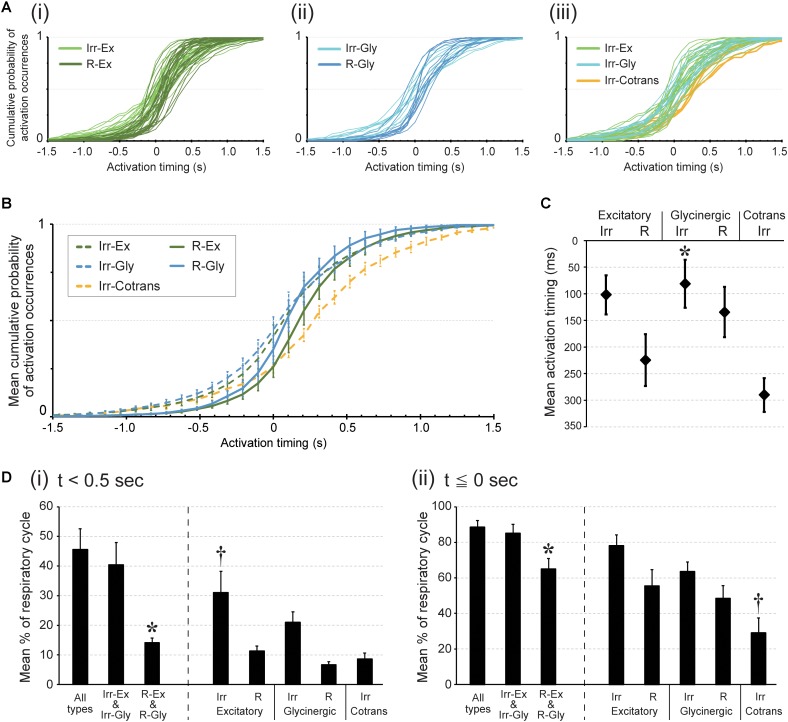
Activation timing during rhythmic burst depends on cell type in the preBötC. Activation timing was detected at the time point of the calcium signal peak. **(A)** Each graph plots cumulative probabilities of activation occurrences (*y*-axis) at each time point around the peak of the LFP (0; *x*-axis) in all inspiratory neurons belonging to respective cell types; (i) irregular (light green) and regular (dark green) types of excitatory neurons: (ii) irregular (pale blue) and regular (dark blue) types of glycinergic neurons: (iii) irregular types of excitatory (light green), glycinergic (pale blue) and Cotrans (yellow) neurons. **(B)** Averaged cumulative probabilities of activation occurrences were plotted at each time point in respective inspiratory cell types (*n* = 5 slices). **(C)** Averaged activation timings in individual inspiratory cell types during rhythmic bursts are shown (^∗^*p* < 0.05 vs. Irr-Cotrans; *n* = 5 slices). **(D)** The graphs show averaged percentages of respiratory cycles in which at least one neuron in the individual cell types were activated (i) before 0.5 s from the peak of LFP and (ii) before and up to the same timing as the LFP peak (i: ^∗^*p* < 0.05 vs. all types group and Irr-Ex and Irr-Gly group, ^†^*p* < 0.05 vs. R-Ex, R-Gly and Irr-Cotrans. ii: ^∗^*p* < 0.05 vs. all types group and Irr-Ex and Irr-Gly group, ^†^*p* < 0.05 vs. Irr-Ex and Irr-Gly; *n* = 5 slices).

In order to follow up our previous results, which showed a stochastic nature of the activation timing of preBötC inspiratory neurons (Oke et al., 2015), we compared the timing of activation among our classified five cell types. As timing parameter for activation, we used the peak of OGB-1 fluorescence fluctuation. Activation timing was determined in more than 100 rhythmic bursts per slice (Slice1, 2, 3, 4, 5; *n* = 147, 219, 219, 114, 193 rhythmic bursts). Irregular-type neurons tended to be activated earlier as compared to Regular-type neurons (**Figures 2A,B**). In all respiratory cycles recorded, 41.8% of activations in Irr-Ex neurons occurred before and up to the same timing as the LFP peak (*t* = 0 s), while in Irr-Gly neurons 45.2% of activations were before and up to *t* = 0 s, in contrast only 25.9% in R-Ex and 35.1% in R-Gly neuron activations occurred that early (**Figure 2B**). Moreover, 10.0% of activation in Irr-Ex and 11.9% in Irr-Gly neurons, but only 4.0% in R-Ex and 4.3% in R-Gly neurons, were detected more than 0.5 s (500 ms) before the LFP peak, when early inspiratory neurons including preinspiratory neurons should be activated (**Figure 2B**). Nevertheless, a neuron that was activated before the LFP peak in one cycle could be activated after the LFP in other cycles (**Figure 2A**). Therefore, we also determined the mean activation timing for each neuron type. The mean activation timing of Irr-Ex and Irr-Gly (Irr-Ex; 102 ± 37 ms, Irr-Gly; 81 ± 45 ms) were earlier as compared to R-Ex (R-Ex; 225 ± 49 ms) (**Figure 2C**). Irr-Cotrans neurons were mainly activated later than the other four cell types as indicated by the mean activation timing (290 ± 32 ms), which was the latest of all cell types, and significantly later than that of Irr-Gly (*p* = 0.0289) (**Figure 2C**).

To identify the leading type of neuron in all cycles, we calculated how often in any respiratory cycles a certain type of neuron was activated before a certain time point, e.g., 0.5 s before the peak of the LFP. In 31.0% of all respiratory cycles, at least one Irr-Ex neuron was actually activated before 0.5 s prior to LFP peak timing, which was significantly greater than for R-Ex, R-Gly and Irr-Cotrans neurons (**Figure 2D** *i, p* = 0.0151, 0.0023, and 0.0085 vs. R-Ex, R-Gly and Irr-Cotrans in each). Activation of one of the neurons from the Irregular-type group, Irr-Ex and Irr-Gly neurons, before 0.5 s from LFP peak was detected in 40.4% of all cycles. This was significantly more often as compared to the Regular-type group, R-Ex and R-Gly neurons (**Figure 2D** *i, p* = 0.04). Activation of one Irr-Ex or Irr-Gly neuron before and up to the same timing as the LFP peak occurred almost all respiratory cycles (85.1%) (**Figure 2D** *ii*).

The cumulative plot of activation timings in Irr-Cotrans neurons is significantly different from those of Irr-Ex, Irr-Gly and R-Gly neurons (left column in **Table 1**), however, activation of Irr-Cotrans neurons occasionally occurred as early as Irr-Ex and Irr-Gly neurons at (*t* = -0.5 s) (9.0%; **Figure 2B**). Furthermore, the activation occurrence of Irr-Cotrans neurons before and up to the peak timing of LFP was detected only in 29.0% of respiratory cycles, which was significantly lower than those of Irr-Ex and Irr-Gly neurons (**Figure 2D** *ii, p* = 0.0017 and 0.0318 vs. Irr-Ex and Irr-Gly). Taken together, Irregular-type neurons, especially Irr-Ex neurons, were activated earlier than Regular-type neurons and Irr-Cotrans neurons.

**Table 1:** Statistical analysis of the distributions.

Cell types compared			Activation timing (Cumulative plots in Figure 2B)	Activation occurrence occupancies at each % rank of activation order (Figure 3A)	Activation occurrence rate in the sequence (Cumulative plots in Figure 3B)
Irr-Ex	vs.	R-Ex	0.157	*p* < 0.001^∗^	0.001^∗^
		Irr-Gly	1.000	0.995	0.995
		R-Gly	0.923	0.009^∗^	0.581
		Irr-Cotrans	0.016^∗^	0.007^∗^	0.014^∗^
R-Ex	vs.	Irr-Gly	0.067	*p* < 0.001^∗^	*p* < 0.001^∗^
		R-Gly	0.392	*p* < 0.001^∗^	0.003^∗^
		Irr-Cotrans	0.289	0.027^∗^	0.674
Irr-Gly	vs.	R-Gly	0.510	0.021^∗^	0.181
		Irr-Cotrans	0.012^∗^	0.031^∗^	0.022^∗^
R-Gly	vs.	Irr-Cotrans	0.005^∗^	*p* < 0.001^∗^	*p* < 0.001^∗^

*Mean values among five slices were examined by pairwised Kolmogorov–Smirnov test*.

### Patterns of Activation Occurrences of Individual Cell Types in the Stochastic Activation Sequence in the PreBötC

**FIGURE 3:**
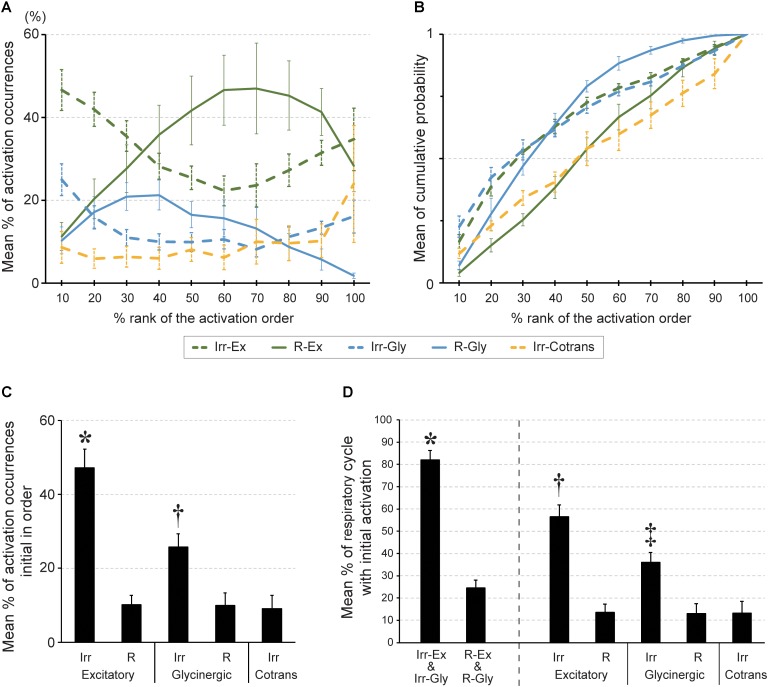
The order of activation during rhythmic burst depends on cell type in the preBötC. **(A)** Averaged percentage of activation occurrences in a certain cell type against those in all cells (*y*-axis) at each percent rank of activation order (*x*-axis). The percentage of activation occurrences was calculated at every bin in the standardized order (bin size: 10% of all neurons). **(B)** Averaged cumulative probabilities of activation occurrences (*y*-axis) were plotted at each percent rank of activation order (*x*-axis) in respective inspiratory cell types. **(C)** Averaged percentage of activation occurrences in a certain cell type against those in all cells at first in the activation sequence (^∗^*p* < 0.01 vs. all other four cell types, ^†^*p* < 0.05 vs. R-Gly; *n* = 5 slices). **(D)** Averaged percentage of respiratory cycles of which a certain cell type of neuron was activated initially in the sequence (^∗^*p* < 0.05 vs. R-Ex and R-Gly group, ^†^*p* < 0.05 vs. R-Ex, Irr-Gly, R-Gly and Irr-Cotrans, ^‡^*p* < 0.05 vs. R-Ex, R-Gly and Irr-Cotrans).

Next, we investigated when the five different cell types fired in relation to other inspiratory neurons. Individual cells were sorted burst-by-burst based on the activation timing in the slice. Then, we calculated how often neurons from each cell type were activated at a given position in the sequence (percentage of activations in a certain cell type/activations in all cells; *n* = 5 slices, **Figure 3A**). At the initial phase (10% and 20% bin) of the activation sequence, Irr-Ex neurons was the major cell type activated. Irr-Gly neurons were also activated early in the sequence and only the plots between Irr-Ex and Irr-Gly neurons were not significantly different in all the combination of cell types (*p* = 0.995, middle column in **Table 1**). To exclude the influence of inequality of the number of inspiratory neurons in individual cell types (**Table 2**), we next calculated when each cell type tended to be activated in the sequence (cumulative probability of activations at a given position in a certain cell type/total activations in the same cell type; *n* = 5, **Figure 3B**). The percentage of activation occurrences at the initial stage (10% and 20% bin) was larger in Irr-Ex than in R-Ex, R-Gly and Irr-Cotrans neurons, which indicates that Irr-Ex neurons actually were activated at an earlier stage. Since the cumulative activation probability of Irr-Gly neurons was almost indistinguishable from Irr-Ex neurons (**Figure 3B** and right column in **Table 1**; *p* = 0.995), the lower percentage of early Irr-Gly neuron activations shown in **Figure 3A** is supposed to be derived from the lower total number of this cell type (**Table 2**). The percentages of activated R-Ex and R-Gly neurons were very low in the first 10% bin but increase later in the cycle (**Figures 3A,B**). As seen from the cumulative probability plot, R-Ex neurons tended to be recruited after R-Gly neurons (**Figure 3B**). Irr-Cotrans neurons were also activated late in the sequence (**Figures 3A,B**).

**Table 2:** Cell type-classified composition ratios of inspiratory neurons.

Slice #	Regular type		Irregular type			Total
	Excitatory	Glycinergic	Excitatory	Glycinergic	Cotrans	
1	3	3	5	2	3	16
	18.8%	18.8%	31.3%	12.5%	18.8%	100%
2	5	1	8	2	1	17
	29.4%	5.9%	47.1%	11.8%	5.9%	100%
3	3	3	4	3	1	14
	21.4%	21.4%	28.6%	21.4%	7.1%	100%
4	9	2	8	2	1	22
	40.9%	9.1%	36.4%	9.1%	4.5%	100%
5	7	2	4	2	0	15
	46.7%	13.3%	26.7%	13.3%	0.0%	100%
Total	27	11	29	11	6	84
	32.1%	13.1%	34.5%	13.1%	7.1%	100%

*All slice were used for analysis in **Figures 2**, **3**. ^∗^Significantly different between indicated two groups*.

These results suggested activation timing depends on the cell type and thus, the cell type defines the framework for the activation sequence of inspiratory neurons in the preBötC. Although Irr-Ex and Irr-Gly neurons together composed only 48% of the total number of inspiratory neurons (**Table 2**), these two cell types were the leading neurons and one of this neurons was found to be activated first in 82% of the respiratory cycles (**Figure 3D**) and 73% of total number of initial activations (**Figure 3C**, Irr-Ex in 47.1% and Irr-Gly in 25.7%). Thus Irregular-type neurons might play a leading role in the generation of rhythmic burst.

## Discussion

In this study, we investigated the activation sequence of respiratory neurons that were defined by molecular markers and activity-patterns to elucidate the functional and structural neuronal network in the microcircuit generating the respiratory rhythm in the preBötC. The current results help to understand earlier observations (Oke et al., 2015) that a subset of inspiratory neurons are activated frequently at the beginning of the activation sequence whereas other neurons are always late. The role of molecularly defined subclasses of neurons (Dbx1^+^, SST^+^, VGAT^+^, and GlyT2^+^ neurons), for the rhythm and pattern generation in the preBötC has been tested *in situ* and *in vivo* using optogenetic techniques and imaging before (Winter et al., 2009; Sherman et al., 2015; Cui et al., 2016; Baertsch et al., 2018), but to our knowledge, this is the first time that the activation sequence of inspiratory neurons in the preBötC is analyzed using genetically coded markers for inhibitory neurons.

**FIGURE 4:**
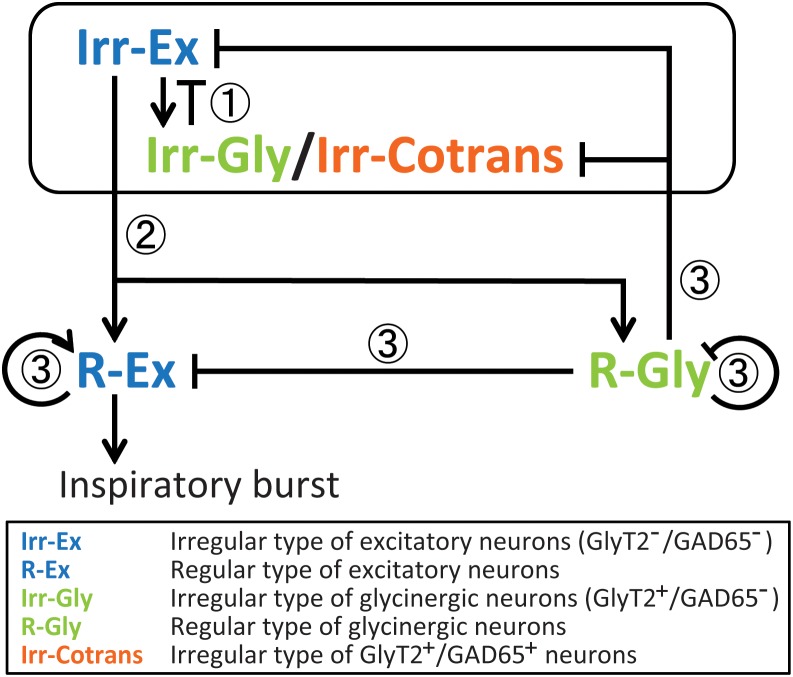
Presumptive inspiratory neuronal network model based on the results of the activation sequence of cell types in the preBötC during rhythmic bursts. In this model, we suppose functional roles of cell types for inspiratory rhythm generation and/or pattern formation in the preBötC. (1) Earlier-phased burstlet: At the initial phase, both Irr-Ex and Irr-Gly/Irr-Cotrans inspiratory neurons are stochastically activated and output from both cell types may influence one another as a mechanism of gain control (Winter et al., 2009; Baertsch et al., 2018). This early activated Irr-Ex and Irr-Gly/Irr-Cotrans neurons might have “pacemaker properties” (Koshiya and Smith, 1999; Morgado-Valle et al., 2010; Kam et al., 2013) and be involved in a low-amplitude preinspiratory component (Kam et al., 2013). (2) Initiation of burstlet: R-Ex and R-Gly inspiratory neurons start to be activated by modulation of Irr-Ex-neuron-mediated excitation and Irr-Gly/Irr-Cotrans-neuron-mediated postsynaptic inhibition. Activated Irr-Ex neurons also contribute to generate the low-amplitude preinspiratory components. (3) Modulation of burst (Gain control): The activated R-Ex neurons induce a high-amplitude inspiratory burst when the power of low-amplitude preinspiratory components, which depends on the amplitude of activation of R-Ex neurons, exceed a certain threshold value. Activation among R-Ex neurons may occur, therefore, this cell type can successively be activated. R-Gly neurons may limit the activities of R-Ex neurons as well as some parts of the activities of Irr-Ex and Irr-Gly/Irr-Cotrans neurons. Even after decrease of the recruitment of R-Gly neurons, glycine released from R-Gly neurons may also inhibit the activity of R-Ex neurons because of its long inhibitory effects.

Based on our data, we propose a network model that accounts for the different activation timing on the five types of inspiratory neurons (**Figure 4**): Two types of neurons with large maxCC, i.e., R-Ex and R-Gly neurons, showed calcium fluctuation waveforms with larger amplitude and longer duration at rhythmic bursts, whereas, three other types of neurons with small maxCC, i.e., Irr-Ex, Irr-Gly and Irr-Cotrans neurons, displayed smaller and shorter calcium signal waveforms. We assume, that coordinated excitatory and inhibitory inputs from these types of neurons are critical for normal rhythm generation and pattern formation in the preBötC.

Irr-Ex and Irr-Gly neurons were activated at the earliest stage of rhythmic bursts, and followed by R-Ex and R-Gly neurons, pointing toward a crucial role for rhythm generation. These results are consistent with the burstlet theory of the Feldman group, (Kam et al., 2013; Feldman and Kam, 2015; Del Negro et al., 2018). The Irr-Ex and Irr-Gly in our study are most likely not regular burster or pacemaker neurons since they are expected to fire only a few action potentials in a cycle [leading to only a low amplitude calcium signal (Winter et al., 2009)]. We postulate that Irr-Ex and Irr-Gly neurons are involved in the low-amplitude preinspiratory components. Furthermore, since the smaller fluctuations of OGB-1 fluorescence in R-Ex neuron were observed during the smaller amplitude of LFP-bursts (**Figure 1F**), R-Ex neurons might also contribute to generation of the low-amplitude preinspiratory components. Therefore, sufficient action potentials firing of R-Ex neurons activated by Irr-Ex neurons can percolate to generate the high-amplitude inspiratory bursts. Considering the wide distribution of activation timings and positions in the activation order seen in Irr-Ex and Irr-Gly neurons (**Figures 2B**, **3B**) and the stochastic change of activation sequence with every respiratory cycle (Oke et al., 2015), Irr-Ex and Irr-Gly neurons could be activated at the earliest phase in the sequence, however, the given position of the neurons in the sequence varied stochastically, cycle-by-cycle. Our assumptions are supported by the previous report that 89% of preBötC inspiratory-modulated neurons generated action potentials during both bursts and burstlets but none of these neurons fired only during burstlets (Kam et al., 2013). Thus, we suggested that postsynaptic excitation from stochastically activated Irr-Ex neurons might be first transmitted to and integrated in R-Ex neurons, which would drive generation of a burst (**Figure 4**).

In this respect, the stochastic sequence of the activation of Irr-Ex neurons before the burst and subsequent activation of R-Ex neurons are supportive for the burstlet theory (Kam et al., 2013; Feldman and Kam, 2015; Del Negro et al., 2018). Further investigation will be required to identify molecular markers, expressed in excitatory inspiratory neurons, especially to discriminate between regular and irregular types.

### Role of Inhibitory Neurons

Irr-Gly-neuron-mediated postsynaptic inhibition might directly give feedback to Irr-Ex, and the inspiratory LFP-bursts could be initiated if the low-amplitude preinspiratory components, which depend on the amplitude of activation of R-Ex neurons, exceed a certain threshold value. We cannot exclude that a subset of Irr-Gly neurons as well as of Irr-Ex neurons might have pacemaker properties, which was observed in a small percentage of inspiratory-modulated neurons firing during both bursts and burstlets (Koshiya and Smith, 1999; Morgado-Valle et al., 2010; Kam et al., 2013). However, since none of the neurons analyzed was the leading neuron in every cycle, we consider this option as less likely.

Our model is consistent with the *in silico* preBötC ‘model’ in which an inhibitory subnetwork influences an excitatory subnetwork to suppress sporadic bursting of excitatory tonic neurons (Lal et al., 2016; Baertsch et al., 2018). Irr-Gly and R-Gly neurons might provide gain control for the excitatory inspiratory neurons (Winter et al., 2009) and, indeed, it was recently shown that co-activation of inhibitory neurons is essential for regulation of refractory periods of dbx^+^ inspiratory neurons (Baertsch et al., 2018). R-Gly neurons are expected to be activated by the same mechanism as also activating R-Ex neurons and to be implicated in a gain control for R-Ex (and also Irr-Ex) neurons to modulate a high-amplitude inspiratory burst (**Figure 4**). Glycinergic neurons also receive inspiratory-related glycinergic inputs (Winter et al., 2009), thus, we presume that R-Gly neurons might limit the activity of Irr-Gly and Irr-Cotrans neurons. Irr-Cotrans neurons were later in the activation sequence compared with other types of neurons even in stochastic activation manner, and may help to terminate respiratory bursts by inhibition of the activity of R-Ex neurons. However, Irr-Cotrans neurons could occasionally be activated as early as Irr-Ex and Irr-Gly neurons (**Figure 2B**). Since the number of this neuron was small, it is difficult to explain this activation property. It is possible that the Irr-Cotrans neurons are functionally simply a part of the Irr-Gly population. Neurons with co-release of GABA and glycine do indeed provide input into respiratory glycinergic neurons in the preBötC (Rahman et al., 2013). Moreover, pharmacological blockade of GABAergic and glycinergic synaptic inhibition induce larger amplitude inspiratory bursts with changes in the interval, including seizure-like bursts (Shao and Feldman, 1997; Baertsch et al., 2018). The low total number of this neurons speaks against dominant role in the core process of rhythm generation. Further analysis is required to identify the role of the co-transmitting neurons.

The burstlet theory for rhythm generation, assumes that the percolation of activity of excitatory neurons is the driving mechanism for the generation of inspiratory burst. The stochastic nature of this process is most likely reflected in the high variability of the activation sequence we found for the Irr-Ex neurons. The fact that different neurons can be in lead of the process adds some redundancy to the network. In pathophysiological conditions, this redundancy may provide a compensatory mechanism to maintain spontaneous respiration output and survival. Even if some neurons from burstlet group turn to be inactive, others form the same cell type can initiate activity and subsequently generate the inspiratory burst.

### Technical Consideration

We defined the activation sequence as the sequence of the time when individual inspiratory neurons functionally give the maximum effect to downstream neurons. The peak of calcium signal reflects the maximum density of action potentials and the neuron can give the greatest influence on the downstream in the neuronal network at the moment. Furthermore, it was technically very difficult to define the ‘real physiological’ starting point (onset timing) of OGB-1 fluorescence fluctuations in our present measurement configuration. Our maximum sampling rate for calcium imaging (about 10 Hz) was too low to detect the starting point or steepest point in OGB-1 fluorescence fluctuation during the rhythmic burst with confidence because of the “noisy” OGB-1 signal. Moreover, inspiratory neurons were not always “silent” between the rhythmic bursts; OGB-1 fluorescence fluctuated even very close to the onset of a burst. Therefore, as a reliable parameter for an activation timing, we used the peak timing of OGB-1 fluorescence fluctuation during a rhythmic burst to evaluate the sequence of the timing when individual neurons can maximally affect other neurons based on their functional properties.

In our study, since we evaluated somatic intracellular calcium dynamics to determine the timing when a neuron fired action potentials during inspiration in the preBötC, we cannot exclude the possibility that dendritic calcium transients contribute to rhythm generation by shaping the inspiratory drive potential. We also did not consider the origin of the action potential in this study.

We could not detect any neurons activated before 0.5 s from the peak timing of LFP in 54% of the respiratory cycles (**Figure 2D** *i*) and before and up to the same timing as LFP peak in 12% of the cycles (**Figure 2D** *ii*). Since the 2-photon laser scanning microscopy system has high spatial resolution, we consider that neurons leading the sequence might be activated at a place where we did not record, in these occasions.

For our analysis it was important to identify bursts in the LFP and not smaller events (Burstlets) as described previously by Kam et al. (2013) using the hypoglossal rootlet as a reference. We minimized the likelihood of including burstlets in our LFP-analysis, by detecting peak timings of calcium fluctuations only when two conditions were concurrently met during rhythmic bursts: (1) the peak value of the integral LFP was larger than 0.8 × standard deviation of LFP signal, and (2) more than half of the inspiratory neurons were activated. Sigh-like big bursts was also excluded from our investigation. During sigh-like big bursts, repeated OGB-1 fluorescence peaks (doublet or triplet) were often observed and some neurons showing simultaneous activation only with sigh-like big bursts. Since sigh rhythm is controlled by neuromodulators, e.g., peptidergic pathways (Tryba et al., 2008; Li et al., 2016), the functional circuitry for sighing might be different from that of eupnea. Thus, revealing the neuronal network for sigh is also important challenge in the future.

Follow up studies with more detailed investigation about the phase-dependent function of respective types of inspiratory neurons are recommended. As an attractive challenge, it also should be tested whether these different activation patterns of cell types during the rhythmic burst can be observed not only in the preBötC slice preparation but also in more intact preparation using *in vivo* preparation. It is expected that these studies can provide further insight into the network connectivity of these neurons for the rhythmic burst of the preBötC.

## Conclusion

We conclude that cell types define a general framework for the activation sequence of inspiratory neurons in the preBötC during spontaneous inspiratory rhythmic bursts, although the individual activation sequence changes stochastically at every respiratory cycle. Our analysis is in line with the concept that a population of irregularly active excitatory neurons initiates a process of excitation and a recruitment of regularly active excitatory neurons as suggest by Kam et al. (2013). However, it also shows that glycinergic neurons are always co-activated with excitatory neurons. Irregular glycinergic neurons are activated as early as irregular excitatory neurons, which is in line with their “pacemaker properties” (Morgado-Valle et al., 2010; Winter et al., 2010). This co-activation appears to be important as a mechanism of gain control (Winter et al., 2009; Baertsch et al., 2018).

## Author Contributions

YOe, YOu, JH, and SH: conception and design of the experiments. YOe and SH: performance of the experiments and writing – original draft and revising it for important intellectual content. YOe, FM, and YOu: data analysis. YOe, FM, YOu, JH, and SH: writing – review and editing, and agreement to be accountable for all content of the work.

## Conflict of Interest Statement

The authors declare that the research was conducted in the absence of any commercial or financial relationships that could be construed as a potential conflict of interest.

## Footnotes

1 http://rsb.info.nih.gov/ij/

## Abbreviations

Cotrans: dual transmitting neuron (GlyT2^+^/GAD65^+^ neuron)
Irr-: irregular type
Irr-Ex: irregular type of excitatory neuron (Irr-GlyT2^-^/GAD65^-^ neuron)
Irr-Gly: irregular type of glycinergic neuron (Irr-GlyT2^+^/GAD65^-^ neurons)
R-: regular type
R-Ex: regular type of excitatory neuron (R-GlyT2^-^/GAD65^-^ neuron)
R-Gly: regular type of glycinergic neuron (R-GlyT2^+^/GAD65^-^ neuron).
