# Corrigendum: Cell Type-Dependent Activation Sequence During Rhythmic Bursting in the PreBötzinger Complex in Respiratory Rhythmic Slices From Mice

**DOI:** 10.3389/fphys.2018.01586

**Published:** 2018-11-09

**Authors:** Yoshihiko Oke, Fumikazu Miwakeichi, Yoshitaka Oku, Johannes Hirrlinger, Swen Hülsmann

**Affiliations:** ^1^Division of Physiome, Department of Physiology, Hyogo College of Medicine, Nishinomiya, Japan; ^2^Department of Statistical Modeling, The Institute of Statistical Mathematics, Tachikawa, Japan; ^3^Department of Statistical Science, School of Multidisciplinary Sciences, The Graduate University for Advanced Studies (SOKENDAI), Tachikawa, Japan; ^4^Carl-Ludwig-Institute for Physiology, Faculty of Medicine, University of Leipzig, Leipzig, Germany; ^5^Department of Neurogenetics, Max Planck Institute of Experimental Medicine, Göttingen, Germany; ^6^Clinic for Anesthesiology, University Medical Center Göttingen, Göttingen, Germany; ^7^Research Center for Nanoscale Microscopy and Molecular Physiology of the Brain, University Medical Center Göttingen, Göttingen, Germany

**Keywords:** preBötzinger complex, inspiratory neuron, inhibitory neuron, rhythmic burst, activation sequence, activation timing, calcium imaging

An author name was incorrectly spelled as **Fumikazu Miwakeich**. The correct spelling is **Fumikazu Miwakeichi**.

In the published article, there was an error in affiliation 3. Instead of “Department of Statistical Science, School of Multidisciplinary Sciences, The Graduate University for Advanced Studies, Hayama, Japan,” it should be “Department of Statistical Science, School of Multidisciplinary Sciences, The Graduate University for Advanced Studies (SOKENDAI), Tachikawa, Japan.”

The authors apologize for these errors and state that this does not change the scientific conclusions of the article in any way. The original article has been updated.

## Conflict of interest statement

The authors declare that the research was conducted in the absence of any commercial or financial relationships that could be construed as a potential conflict of interest.

